# Second Harmonic Generation Imaging of Collagen in Chronically Implantable Electrodes in Brain Tissue

**DOI:** 10.3389/fnins.2020.00095

**Published:** 2020-07-07

**Authors:** Corinne R. Esquibel, Kristy D. Wendt, Heui C. Lee, Janak Gaire, Andrew Shoffstall, Morgan E. Urdaneta, Jenu V. Chacko, Sarah K. Brodnick, Kevin J. Otto, Jeffrey R. Capadona, Justin C. Williams, K. W. Eliceiri

**Affiliations:** ^1^Laboratory for Optical and Computational Instrumentation, Department of Biomedical Engineering, University of Wisconsin, Madison, WI, United States; ^2^Weldon School of Biomedical Engineering, Purdue University, West Lafayette, IN, United States; ^3^J. Crayton Pruitt Family Department of Biomedical Engineering, University of Florida, Gainesville, FL, United States; ^4^Department of Neuroscience, University of Florida, Gainesville, FL, United States; ^5^Department of Biomedical Engineering, Case Western Reserve University, Cleveland, OH, United States; ^6^Advanced Platform Technology Center, Louis Stokes Cleveland Department of Veterans Affairs Medical Center, Cleveland, OH, United States; ^7^Morgridge Institute for Research, Madison, WI, United States; ^8^Department of Medical Physics, University of Wisconsin, Madison, WI, United States

**Keywords:** second harmonic generation, collagen, glial scar, imaging, implantable device

## Abstract

Advances in neural engineering have brought about a number of implantable devices for improved brain stimulation and recording. Unfortunately, many of these micro-implants have not been adopted due to issues of signal loss, deterioration, and host response to the device. While glial scar characterization is critical to better understand the mechanisms that affect device functionality or tissue viability, analysis is frequently hindered by immunohistochemical tissue processing methods that result in device shattering and tissue tearing artifacts. Devices are commonly removed prior to sectioning, which can itself disturb the quality of the study. In this methods implementation study, we use the label free, optical sectioning method of second harmonic generation (SHG) to examine brain slices of various implanted intracortical electrodes and demonstrate collagen fiber distribution not found in normal brain tissue. SHG can easily be used in conjunction with multiphoton microscopy to allow direct intrinsic visualization of collagen-containing glial scars on the surface of cortically implanted electrode probes without imposing the physical strain of tissue sectioning methods required for other high resolution light microscopy modalities. Identification and future measurements of these collagen fibers may be useful in predicting host immune response and device signal fidelity.

## Introduction

Multiphoton microscopy is now a widely adopted brain imaging method, and can be used to monitor *in vivo* neural activity with single spine resolution (Knott et al., 2006; Svoboda and Yasuda, 2006; Kerr and Denk, 2008; Holtmaat et al., 2009; Ozbay et al., 2018). As a non-linear modality, multiphoton offers spatial confinement to the focal region in scattering brain tissue and allows deep, high-resolution optical sectioning of live brain or thick sections *in vitro* (Kobat et al., 2011). Multiphoton microscopy can generate both fluorescence and second harmonic generation (SHG) as simultaneous contrast mechanisms, which provide complementary information regarding tissue structure and function, as well as orientation, polarization, and symmetry properties of chiral proteins (Zoumi et al., 2002; Belluscio, 2005; Provenzano et al., 2010; Chen et al., 2012). SHG generates its intrinsic contrast from the interaction of light with non-centrosymmetric structures such as collagen I, collagen II, and myosin (Roth and Freund, 1979; Plotnikov et al., 2006; Chen et al., 2012). SHG is a coherent optical process during which two photons combine and emit a single photon with visible light. As such, SHG imaging offers many of the same benefits of traditional multiphoton microscopy. SHG can be used for high resolution, deep imaging of tissues, allowing a depth penetration of up to ∼500 μm. The triple-helix structure of fibrillar collagen permits visualization up to 0.2–0.3 μm resolution with little to no tissue damage, and does not require the use of fluorescent labels, stains, or genetically modified species (Williams et al., 2005; Li et al., 2011; Chen et al., 2012; Mostaço-Guidolin et al., 2017). While the current study was performed in *ex vivo* brain slices, SHG can also be used *in vivo* to observe changes over time (Zoumi et al., 2002; Dilipkumar et al., 2019). Though the phenomenon of SHG was first demonstrated in biological tissues over three decades ago, and is easily observed with the appropriate filter, it remains an underutilized modality by those already using multiphoton microscopy to image brain-implanted devices *in vivo* and *in vitro* (Freund and Deutsch, 1986; Chen et al., 2012). One factor might be that the most common application for SHG imaging is examining fibrillar collagen and the role of collagen in the brain is still emerging (Shearer and Fawcett, 2001; Heck et al., 2003).

Extracellular matrix (ECM) molecules in the unwounded brain occupy up to 20% of adult brain volume and are characterized by long, linear polysaccharide glycosaminoglycans such as chondroitin sulfate and hyaluronan, while fibrillar collagen is notably absent (Syková and Nicholson, 2008; Miyata and Kitagawa, 2017). Brain ECM exists in diffuse forms found throughout the neuropil and perisynaptic spaces and condensed forms called perineuronal nets (PNNs) that form lattice-like structures around subpopulations of neurons (Miyata and Kitagawa, 2017). While glycosaminoglycans in brain ECM were previously considered non-specific physical barriers to neural regeneration, recent studies have proposed that ECM molecules actively regulate neuronal function through specific interactions with their binding partners (Miyata and Kitagawa, 2017). Though non-fibrillar types of collagen have been observed in healthy brain tissue and have been shown to be necessary for proper function (Seppänen et al., 2007; Hubert et al., 2009; Su et al., 2010) the brain does not typically show the same patterns or abundance of fibrillar collagen (Rauch, 2007; Fox, 2008). However, early experiments suggest the existence of fibroblasts and fibrillar collagens of types I, III, IV, and V within wound areas in the brain (Berry et al., 1983; Maxwell et al., 1984, 1990).

When a penetrating lesion is made in the adult rat cerebral hemisphere, the initial hemorrhagic reaction is followed by invasion of blood-borne macrophages and fibroblasts from the adjacent connective tissue into the lesion lumen, resulting in collagen fibril and basement membrane formation (Berry et al., 1983; Maxwell et al., 1984). The first responders after electrode insertion are microglia, the macrophage lineage cells of the brain, which begin their activation within minutes of injury and show increased density within 24 h (Davalos et al., 2005; Nimmerjahn et al., 2005; Kozai et al., 2015). Reactive astrocytes peak within the first week following injury, and within approximately three to 4 weeks form a compact, collagen-containing sheath around any foreign bodies that remain (Biran et al., 2005). Glial scar formation around chronically implanted electrodes is a reactive, cellular process with rapidly changing cell population dynamics that include perivascular-derived fibroblasts, pericytes, ependymal cells, and phagocytic macrophages (Adams and Gallo, 2018). Immunohistological labeling of various populations of activated fibroblasts and astrocytes surrounding the lesion core requires histological sectioning, a process that frequently causes artifactual damage to electrodes and surrounding tissue under analysis. While others have reported that the fibrotic scar is replete with collagen (Shearer and Fawcett, 2001), the advantages it offers as an endogenous marker of glial scar formation imageable by SHG is largely unrecognized even by those already characterizing rodent brain tissue around chronically implanted electrodes by multiphoton microscopy.

Established histological methods for identifying collagen in tissue include immunohistochemical staining for collagen types I and II as well as non-specific anionic dye procedures such as Van Gieson’s stain, Masson’s Trichrome, and Sirius Red. Anionic dyes stain collagen by reacting acid groups with the basic groups of collagen, and standard method protocols specify tissue section thicknesses of 5 μm in paraffin sections to permit dye penetration into the tissue. In Sirius Red staining, the elongated axis of dye molecules are attached parallel to the collagen fiber, resulting in enhanced birefringency and specificity when combined with polarized light detection methods. A traditional transmission pathology microscope fitted with linear polarizers or more specialized instrumentation such as the liquid crystal based PolScope perform optimally with standard histological tissue section thicknesses between 5 and 10 μm, so that light can effectively pass through the specimen for phase-shift contribution to contrast in the final image.

Ischemia of a resected specimen before fixation for immunohistochemistry can result in degradation of protein, RNA, and DNA as well as activation of tissue enzymes and autolysis, and small variations in ischemic time can be a crucial factor affecting IHC results. Thick tissue sections can produce higher background signals as can frozen sections, and soluble antigen may be diffused out during the process of IHC prior to fixation. Immunohistochemical protocols for the anticollagens I and II antibodies specify 30–40 μm thick frozen tissue sections, and instruct cutting thinner sections for greater permeation of antibody. These methods typically result in electrode shattering or tearing and separation of the electrode from the tissue for industry standard silicon-based NeuroNexis probes with thicknesses of 15 μm or greater.

None of the methods for imaging collagen described above offer the depth (100s of microns) and non-invasiveness of SHG to image implanted electrode surfaces in histological thick sections 300–500 μm as described in this experiment. SHG is highly specific to the non-centrosymmetric structure of fibrillar collagen, offers high resolution, good signal-to-noise ratio, and ability to work non-destructively on stained and unstained tissues. Unlike fluorescence, SHG suffers no inherent photobleaching or toxicity and does not require exogenous labels. Unlike polarization microscopy, SHG provides intrinsic confocality and deep sectioning in complex tissues.

Previous antibody-based studies have shown collagen I deposition around penetrating neural implants (Kim et al., 2004). However, label-free, high resolution SHG based imaging of the collagenic scar around implanted neural electrodes has not been demonstrated. In this method validation study, multiple penetrating electrodes types harvested at different times post-implantation were imaged post-mortem by SHG to confirm the presence of collagen fiber deposition around the device. We demonstrate collagen fibrils associated with implanted tissue not found in normal brain tissue. Label-free measures of collagen fibers around intact implanted electrodes may be useful in predicting host immune response to various electrode device designs and may also predict signal fidelity of the device.

## Materials and Methods

*Ex vivo* SHG images of brain slices in the backward direction were collected through a Nikon 20× water-dipping objective (1.0 NA) at 890 nm excitation. A dichroic cube filter set (Chroma Technologies, Bellow Falls, VT, United States) containing two band-pass emission filters SHG (445/40 nm) and flavin adenine dinucleotide (FAD) (592/100 nm) were used in an imaging system consisting of a multialkali photomultiplier detector (Hamamatsu, Shizuoka, Japan) on a Bruker Ultima IV (Bruker FM, Middleton, WI, United States) multiphoton microscope equipped with an Insight ultrafast laser (Spectra Physics, Santa Clara, CA, United States). A motorized stage was used to automatically collect images at tiled x/y locations throughout the brain sections. Tiled images were stitched together using FIJI’s grid/collection stitching plugin (Preibisch et al., 2009).

The power at the back aperture of the objective was ∼7 mW. For 890 nm, 1.0NA, at these powers the lateral resolution is 380 nm and axial resolution ∼4 um. Imaging parameters were optimized by starting with low Pockels cell values using a range check LUT to display intensity scale for an image to ensure optimal saturation to prevent photobleaching and phototoxicity. The images from the Ultima are digitized to 12 bits, which means that the input channel data intensity scale ranges from 0 (no signal) to 4095 (saturated signal). In a black and white LUT, values of 0 are usually represented as pure black and values of 4095 are usually represented as pure white. Since the computer only has 256 gray levels, a function or LUT is used to define the display intensity scale. If these 256 display gray levels are used to display the full range of 4096 intensity levels then each display gray level is equal to 16 PMT data intensity levels. Photomultiplier tube values on multialkali PMTs were 650–700.

To help validate the SHG based observations, antibody staining on a horizontal electrode was done. Primary antibody staining was done with 1:750 Chicken anti-GFAP (EMD Millipore, AB5541) and 1:300 Rabbit anti- Collagen I (Novus Biologicals, NB600-408). Secondary antibodies used were 1:500 Goat Anti-Rabbit IgG H&L (FITC) (Abcam, AB6717) and 1:1000 Alexa-fluor-633 goat anti-chicken (Invitrogen). The samples were mounted with Vectashield mounting medium with DAPI (Vector Laboratories, Burlingame, CA, United States) and imaged on a Leica DMi8 fluorescence microscope (Leica Microsystems, Wetzlar, Germany) with a 10x 0.4 NA air immersion dry objective (Leica Microsystems, Wetzlar, Germany) and the following filter cubes CFP (Ex. 426–446, Em. 435–485), GFP (Ex. 450–490, Em. 500–550) and Y3/RFP (Ex. 532–558, Em. 570–640). The fluorescence images were registered with the SHG images from the same slide with fine-structures registration approach (BUNWARPJ, FIJII). A mask from SHG was used to see if all the pixels are co-registered.

Off-stoichiometry thiol-enes-epoxy (OSTE+) polymer probes were fabricated in the laboratory of Marting Bengtsson at Lund University, implanted in mice and harvested as previously described by Lee et al. (2017) following Institutional Animal Care and Usage Committee (IACUC) guidelines at the University of Florida. OSTE+ Hard, OSTE+ Soft, polyimide, and silicon electrodes had dimensions of 250 μm wide and 3 mm long with a tapered tip of approximately 18° in angle. Thicknesses were: 21.3 ± 1.0 μm (polyimide), 23.5 ± 2.1 μm (OSTE+Hard), and 22.4 ± 2.1 μm (OSTE+Soft) (mean ± standard deviation, *N* = 15). Probes were cortically implanted in mice and harvested at 4 and 6 weeks respectively. Mouse brains were lightly embedded with optimum cutting temperature (OCT) compound (Sakura Finetek, Netherlands) and sliced into 25 μm horizontal sections with the retained probes. More common varieties of NeuroNexus silicon probes from implanted rat brains were imaged at various time points as previously described (Woolley et al., 2013). Intact single shank NeuroNexus probes (249 μm width, 15 μm thickness, *N* = 3; 132 μm width, 15 μm thickness, *N* = 2), single shank bare silicon probes (132 μm width, 15 μm thickness, *N* = 3), and quadruple shank bare silicon probes (132 μm width, 15 μm thickness, *N* = 2) were collected within coronal slices of rat brain harvested between 53 and 177 days prior to sacrifice using the device capture technique described by Woolley et al. (2013) and sectioned between 350 and 450 μm.

## Results

**FIGURE 1:**
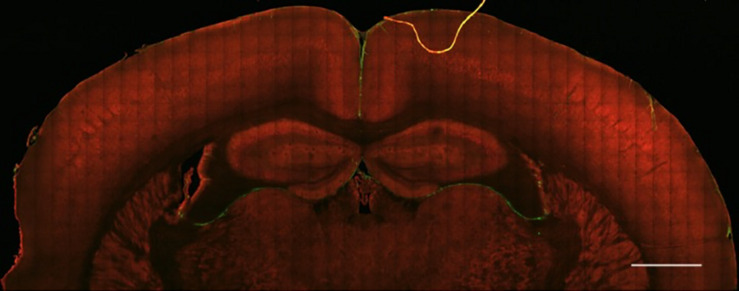
Healthy brain tissue is largely devoid of fibrillar collagen. Second Harmonic Generation (SHG) imaging of mouse brain tissue shows virtually no fibrillar collagen (green, 890 nm excitation 445/40 nm emission) within the parenchyma. Collagen fibers can be seen surrounding the cortex and within and between ventricles. Multiphoton induced autofluorescence (red, 890 nm excitation, 592/100 nm emission, likely FAD) was recorded to observe gross anatomical features of the tissue. Scale bar = 1 mm.

**FIGURE 2:**
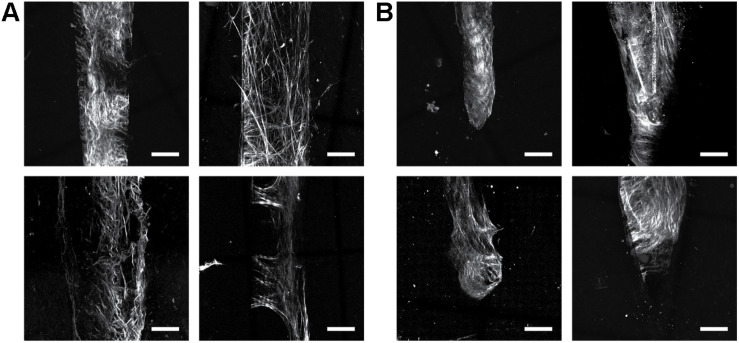
Collagen fibers encapsulate indwelling neural devices. Abundant collagen fibers are shown with SHG imaging (890 nm excitation 445/40 nm emission) of a 132 um NeuroNexus silicon device, implanted for 56 days, captured within a coronal slice of rat brain tissue. **(A,B)** Quantify and visualize, respectively, that collagen fibers are only observed on the surface of the device, not in the surrounding parenchyma. **(C)** Magnifies a 500 um × 200 um segment of the image, clearly showing fibers encircling the device as well as extending along the length of the device. When the depth of the collagen within the imaged z-stack is encoded as color (shallow z-depth, surface of tissue slice = white; deep z-depth, interior of tissue slice = indigo), circumferential fibers can be observed both above below longitudinal fibers.

**FIGURE 3:**
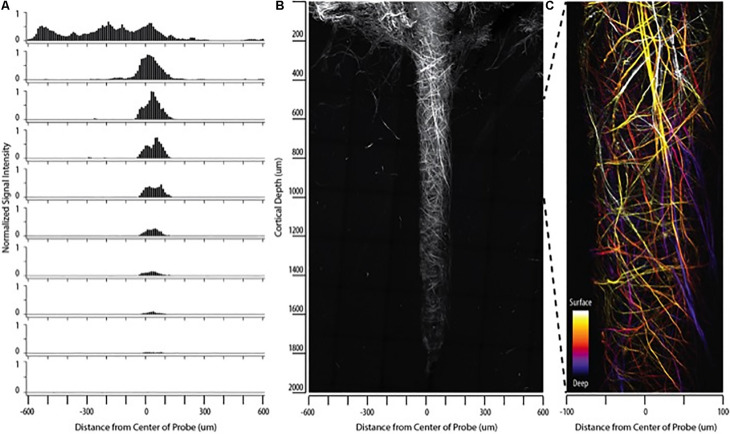
Collagen fibers observed with SHG imaging match the geometry of implanted neural devices independent of probe size/type in silicon NeuroNexus probes. Although the manifestation of fibrillar collagen varied, fibers consistently conformed to the implant shape, encompassing both the **(A)** shank and the **(B)** tip of the device. Electrodes were implanted between 53 and 177 days prior to sacrifice. Scale bar = 50 um.

**FIGURE 4:**
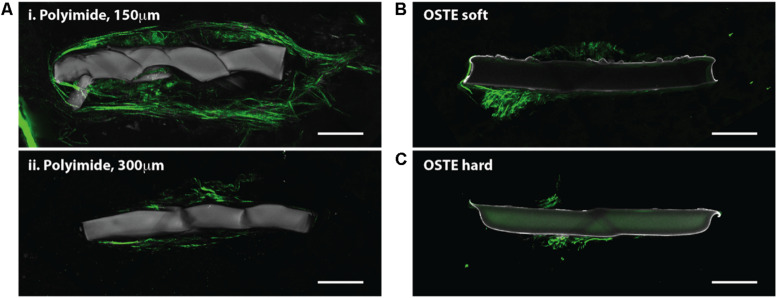
Horizontal slices through implanted probes show collagen deposition. SHG imaging (green, 890 nm excitation 445/40 nm emission) shows collagen fibers around neural probes, independent of the indwelling device material. Multiple slices through the same polyimide device show that the collagen fibers are most abundant close to the cortical surface **(Ai)** but are still observable in deeper cortical regions **(Aii)** Collagen fibers were also observed on and around neural probes made of OSTE soft **(B)** and OSTE hard **(C)** materials. Autofluorescence from the implanted device is shown in gray (890 nm excitation, both 445/40 and 592/100 nm emission). Scale bar = 50 microns.

**FIGURE 5:**
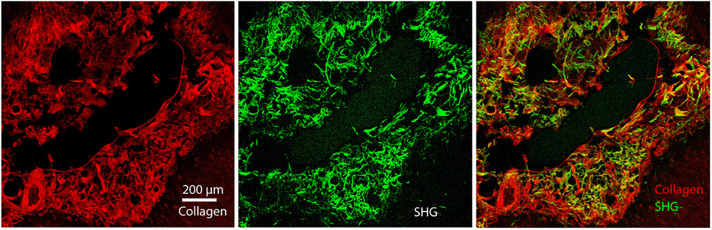
Immunohistochemistry of Collagen I overlaid with GFAP of the astrocytic glial scar in chronic horizontal slices. We re-registered the two files with fine-structures registration (BUNWARPJ, FIJI). We used a mask from SHG to see if all the pixels are co-registered. We found 88% of the SHG pixels belong to the collagen 1 antibody staining. Scale bar = 200 microns.

We report the direct observation of high-resolution collagen fibers encapsulating intact, indwelling silicon NeuroNexus neural devices in thickly sectioned (350–450 μm) rat coronal slices (*N* = 10 for all varieties of NeuroNexus probes) that contrasts with the absence of non-fibrillar collagen in the unwounded brain (Figures 1, 2). In Figure 2, abundant collagen fibers within a substantial glial scar are shown with SHG imaging (890 nm excitation 445/40 nm emission) of a NeuroNexus silicon device, implanted for 8 weeks, sectioned at 400 μm, and captured within a coronal slice of rat brain tissue. Collagen fibers on the surface of the silicon device are shown in Panels A and B. Panel C magnifies a 500 μm × 200 μm segment of the image, clearly showing fibers encircling the device as well as extending along the length of the device. When the depth of the collagen within the imaged z-stack is encoded as color (shallow z-depth, surface of tissue slice = white; deep z-depth, interior of tissue slice = indigo), circumferential fibers can be observed both above and below longitudinal fibers. Collagen fibers that match the geometry of implanted silicon NeuroNexus devices (time of implantation ranged from 53 to 177 days) can be observed with SHG imaging independent of probe size and shank number (Figure 3). Imaging 25 μm histological sections of mouse brain tissue harvested at 4 and 8-week timepoints proved more difficult due to the artifacts induced by tissue processing required by the criteria of that study (Lee et al., 2017). In these samples, the softer electrodes were physically cross-sectioned by the 25 μm sectioning processing method. Though collagen fibers were still observable, the degree of apparent collagen deposition was decreased in samples processed in this manner (Figure 4). When tissue from animals implanted with silicon probes was cross-sectioned at 25 μm, the probes shattered and pulled away from the tissue, disallowing observation of collagen fibers. Given the fragile nature of the tissue and electrodes it was challenging to do any sort of antibody validation on most of these samples, few survived the process. However, we were able to take one of the horizontal electrodes and stain it for collagen I and GFAP (Figure 5). We used a mask from SHG to see if all the pixels are co-registered. We found 88% of the SHG pixels belong to the collagen 1 antibody staining.

## Discussion

Understanding glial scar formation is fundamental to improving biocompatibility of chronic implanted electrodes. Scar borders are formed primarily by newly proliferated astrocytes and glia that surround and recruit inflammatory and fibrotic cells into discrete areas that are separated from adjacent tissue containing viable neurons (Wanner et al., 2013). Our observations of fibrillar collagen on the surface and perimeter of chronically implanted electrode probes in rat and mouse, correspond with the glial scar and suggest a correlative signal that may be used to assess and quantify wounding in the brain caused by chronically implanted electrodes. This signal can be evaluated in both *in vivo* and thickly sectioned post-mortem coronal sections, without the addition of extrinsic stains or fluorescence. This study offers a new method to assess and potentially quantify scarring around a chronically implanted electrode. While fibrillar collagen has not been previously reported on the surface of electrodes in conjunction with glial scar formation, it appears to coincide with glial scar location.

Glial scars provide a biochemical and mechanical barrier to neuronal generation and result in tissue softening in the cortex after injury (Moeendarbary et al., 2017). Blood brain barrier (BBB) leakage, astrogliosis, and tissue remodeling correlate with a reduction in silicon microelectrode array recording performance, increases in microelectrode impedance and loss of neuronal recording attributed to the encapsulating brain tissue response (Nolta et al., 2015). Changes to the tissue microenvironment surrounding the device can dramatically impact electrochemical and electrophysiological signal sensitivity and stability over time (Williams et al., 1999). Glial cells form tight junctions with each other to create a glial sheath, which in combination with collagen along the length of the electrode can form a diffusion barrier that limits transmission of ions as well as overflow of neurotransmitters through the extracellular space (Roitbak and Syková, 1999). In general, this increase in impedance is observed over the first 2 weeks following insertion, before stabilizing (Williams et al., 1999; Kozai et al., 2014). Furthermore, neuronal cell death and degeneration of neurites can occur within 150 μm of the device over the first 4 weeks (Biran et al., 2005; Kozai et al., 2014, 2015). The long-term utility of neural devices depends on the severity of this tissue reaction, with chronically implanted devices becoming less reliable over time (Williams et al., 1999; Polikov et al., 2005; Barrese et al., 2013; Sommakia et al., 2014).

Research also suggests that a mechanical mismatch between the softer brain tissue and industry standard silicon electrodes may induce cellular sheath formation, particularly near the tip and edges of the probe where the highest elevated local strains occur (Lee et al., 2017). Strain–stress caused by neural implant rigidity and the brain’s micromotion may exacerbate the foreign body response (FBR). Flexible nanocomposite probes (12 MPa) induce significantly less neuroinflammatory response than standard silicon probes and polyvinyl acetate (PVAc)-coated silicon probes (Harris et al., 2011; Nguyen et al., 2014) and flexible penetrating devices have been shown to have comparatively long-term electrophysiological characteristics in the CNS. Lee et al. (2017) observed a significant reduction in the fluorescence intensity of biomarkers for activated microglia/macrophages and BBB leakiness around three types of soft polymer probes compared to silicon probes at 4 and 8 weeks post-implantation, suggesting that the mechanical compliance of neural probes can mediate the degree of FBR.

Reactive astrocytes are believed to be the main contributors of the molecular cues that drive glial scar formation in the wounded brain (Ridet et al., 1997; Heck et al., 2003). When astrocytic cell lines were developed with a range of abilities to promote or inhibit neurite outgrowth, the most inhibitory of these cell lines, Neu7, was correlated with fibrillar collagen production (Heck et al., 2003). The glial scar may impede electrophysiology measurements by directly altering the impedance or ionic microenvironment, or by simply increasing the physical distance between the neurons and the recording contacts. Identifying and modulating potentially inhibitory molecules or physical barriers in the ECM will be critical to developing interventions that allow axons to regenerate beyond the glial scar (Silver and Miller, 2004; Fitch and Silver, 2008; Cregg et al., 2014).

SHG can penetrate 100s of microns in brain tissues, making it an appropriate technique for imaging without risking shattering the sample or tearing the tissue during sectioning as occurred in the brain samples harvested in mice for Lee et al. (2017). The most commonly used laser for SHG imaging offers average performance for multiphoton imaging in the brain, the Nd:YVO4 (532 nm; 5–18 W) pumped Ti:sapphire oscillator, that has tuning ranges of ∼700–1,000 nm, repetition rates of ∼80 MHz, average powers of 1–2 W and pulse widths of ∼100 fs, which correspond to a bandwidth of about 10 nm full width at half-maximum (FWHM) (Chen et al., 2012). SHG is not a resonant process, and the choice of excitation wavelength in terms of signal intensity is thus not crucial (Chen et al., 2012). 900-nm excitation is a good compromise between imaging depth, viability and Ti:sapphire performance (Chen et al., 2012). A short wave pass (SWP) dichroic mirror following the laser is necessary for background-free SHG detection, as residual pump (532 nm) can co-propagate with Ti:sapphire through the entire microscope path to the detectors.

SHG can be used to visualize collagen to improve our understanding of how ECM components impact and participate in the foreign body response to implanted neural devices. While this study demonstrated that collagen coincides with glial scar location, a true comparison of the FBR in electrode material will require normalized distribution of tissue processing methods, electrode types, and rodent species. We demonstrate high-resolution, in-depth imaging of fibrillar collagen on the surface of the implants coinciding with glial scar location in intact electrodes in thickly sectioned samples (350–450 μm) without artifacts typically induced by histological sectioning. Future studies should investigate whether the material type or composition of the electrode affects the collagen response. This imaging tool could enable rapid evaluation of new probe designs and therapies aimed at reducing the formation of the glial scar to ultimately improve the chronic performance of implanted neural devices.

## Data Availability Statement

All datasets generated for this study are included in the article/supplementary material.

## Ethics Statement

The animal study was reviewed and approved by University of Wisconsin at Madison.

## Author Contributions

CE, KW, JG, HL, AS, MU, and JC carried out the measurements. JW, CE, and KE conceived the experiment and KE supervised the project. All authors participated in designing the research and writing the manuscript.

## Disclaimer

The contents of this paper do not represent the views of the U.S. Department of Veterans Affairs or the United States Government.

## Conflict of Interest

The authors declare that the research was conducted in the absence of any commercial or financial relationships that could be construed as a potential conflict of interest.
